# Design and Optimization of the Dual-Mode Lamb Wave Resonator and Dual-Passband Filter

**DOI:** 10.3390/mi13010087

**Published:** 2022-01-05

**Authors:** Tiancheng Luo, Yan Liu, Yang Zou, Jie Zhou, Wenjuan Liu, Guoqiang Wu, Yao Cai, Chengliang Sun

**Affiliations:** The Institute of Technological Sciences, Wuhan University, Wuhan 430072, China; luotiancheng95@whu.edu.cn (T.L.); liuyan92@whu.edu.cn (Y.L.); yang_zou@whu.edu.cn (Y.Z.); zhoujie_ist@whu.edu.cn (J.Z.); lwjwhu@whu.edu.cn (W.L.); wuguoqiang@whu.edu.cn (G.W.)

**Keywords:** 5G communications, radio frequency (RF) integration, Lamb wave resonator, interdigital electrodes, dual modes, dual-passband filter

## Abstract

Radio frequency (RF) filters with multiple passbands can meet the needs of miniaturization and integration for 5G communications. This paper reports a dual-mode Lamb wave resonator (DLWR) and a dual-passband filter based on DLWRs. The DLWR consists of a piezoelectric film and two interdigital electrode (IDT) arrays with different thicknesses, which leads to the coexistence of two main modes in the resonator. The resonance frequencies of the two modes can be adjusted separately by changing the thicknesses of the IDTs, which greatly satisfies the requirements of the dual-passband filter. Four DLWRs with different electrode configurations are designed, and the influences of the periodic length and thicknesses of the IDTs on the performance of the DLWR are studied. When the thickness of the piezoelectric layer is 0.75 μm and the two thicknesses of the IDTs are 0.1 μm and 0.3 μm, the resonance frequency of the second main mode is 1.27 GHz higher than the resonance frequency of the first main mode in the DLWR. Furthermore, a dual-passband filter based on the proposed DLWRs is demonstrated with an insertion loss less than 1 dB and a band rejection of about 15 dB. Moreover, two passbands at 2.45 GHz and 3.88 GHz with bandwidths of 66 MHz and 112 MHz, respectively, are achieved. The presented DLWR shows a potential application that can be used to obtain RF filters with adjustable dual passbands.

## 1. Introduction

A highly integrated radio frequency (RF) front-end module is in demand with the rapid commercialization of fifth generation (5G) technology [1,2,3,4]. RF filters based on piezoelectric resonators play an important role in the front-end module. Moreover, the requirements of miniaturization and integration in filters are desired to achieve low-energy consumption and a light weight. Reducing the area of resonators is a choice, but the small active area may affect the performance of the resonator. Scaling down the dimensions of microelectromechanical system (MEMS) resonators can bring severe power handling and nonlinearity issues, as well as decrease the coupling coefficient and deteriorate the quality factor (*Q*) [5]. A multi-passband filter based on multi-mode resonators can realize the function that needs multiple single-passband filters. Integrating multiple passbands into a filter is an effective method to reduce the dimension and number of electronic devices in the RF front-end module without sacrificing the performance of the filters [6]. In addition, dual-mode resonators also have important applications in sensors [7] and other fields [8,9].

Many efforts are devoted to design and implement multiple passband filters. L. C. Tsai et al. [10] proposed a dual-band bandpass filter, which can be realized by cascading band-pass and band-stop filters. A superior dual-mode resonator using a seven-layer Mo/SiO_2_ Bragg reflector and the c-axis-tilted AlN was realized by C. J. Chung et al. [11]. John D. Larson et al. [12] proposed a dual-passband filter based on piezoelectric resonators via two piezoelectric layers with oppositely grown orientation. A theoretical analysis of thickness-longitudinal and thickness-shear dual-mode resonators based on c-tilted ZnO and AlN films was presented by L. Qin et al. [13,14]. Moreover, Y. Zou et al. [15] further studied the dual-mode resonators with c-tilted films using Finite Element Analysis (FEA) and proposed a potential application for the dual-passband filter.

Lamb wave resonators (LWRs) have been proposed [16,17,18], the frequency of which can be defined by not only the thickness of the piezoelectric film but also the longitudinal and lateral dimensions of their interdigital electrodes (IDTs) [19,20,21]. LWRs based on aluminum nitride (AlN) possess advanced and attractive properties for enabling the next-generation single-chip radio frequency front-end module [22] because of the advantages, including lithographically defined resonance frequency, high phase velocities (*v*_p_), small motional impedances (*R*_m_) and a moderate electromechanical coupling factor (*k*^2^) [23]. A Lamb wave resonator utilizing an aluminum nitride (AlN) plate with biconvex edges to enhance the quality factor is demonstrated by Chih-Ming Lin et al. [24], and the measured frequency response of a 491.8 MHz AlN Lamb wave resonator with biconvex edges yields a *Q* of 3280, which represents a 2.6× enhancement. However, dual-passband filters based on LWRs are rarely reported.

In this paper, we propose the design of a dual-mode Lamb wave resonator (DLWR) and a RF dual-passband filter based on DLWRs. The DLWR consists of a piezoelectric film and two IDT arrays with different thicknesses. Moreover, DLWRs with four different electrode configurations are analyzed. The influences of IDT thicknesses and IDT pitch (periodic length) on resonance frequencies (*f*_s_), quality factor (*Q*), effective electromechanical coupling coefficient ($K_{eff}^{2}$) and spurious modes are investigated. The most important factor is that the two resonance peaks of the DLWR can be adjusted separately by changing the thicknesses of the IDTs. Moreover, by using DLWRs, filters with two adjustable passbands can be obtained. This paper provides a novel method to achieve dual-mode resonators and dual-passband filters.

## 2. Modeling and Analysis

### 2.1. Structure of Resonators

**Figure 1:**
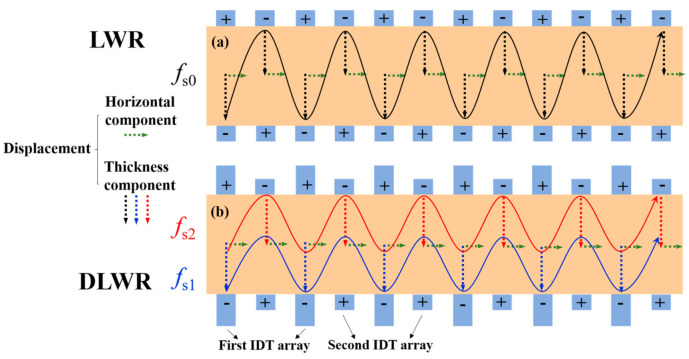
Structural design and displacements of (**a**) LWR and (**b**) DLWR.

The difference between LWRs and DLWRs is the design of the IDTs. Figure 1 shows device structures of a conventional LWR and the proposed DLWR. Acoustic waves in LWRs and DLWRs are both excited by IDTs. The thickness of all IDTs in LWRs is the same, and there is a significant correlation between the resonance frequency (*f*_s0_) and the thickness of the IDTs. The mode is identified as Lamb wave when the shear displacement is negligible compared with the other two displacement components (longitudinal and shear vertical) [25]. The displacement of LWRs can be equated to the superposition of the thickness direction and the horizontal direction even though the propagation is quite complicated [26]. As shown by the black dotted line in Figure 1a, the displacement magnitude of the thickness direction of all IDTs in LWRs is the same at the resonance frequency. Considering the thickness direction and the horizontal direction, the black solid line represents the IDTs’ total displacement of the LWR at the resonance frequency.

However, the displacement magnitude of DLWRs is significantly different from that of LWRs when the IDTs are split into two equidistant arrays. The first IDT array in DLWRs is thicker, and the second IDT array is thinner. Moreover, the top and bottom IDTs are alternately supplied with voltages of different polarities. The red and blue curves in Figure 1b represent the electrode displacements of the two main resonance modes, respectively. The resonance frequencies of the two modes are different. The resonance frequency of the first main mode (*f*_s1_) in DLWRs is lower, which depends on the thickness of the thicker IDT array, and the displacement magnitude of the thicker IDT array is large while that of the thinner IDT array is small at *f*_s1_. Similarly, the resonance frequency of the second main mode (*f*_s2_) in DLWRs is higher, which depends on the thickness of the thinner IDT array, and the displacement magnitude of the thinner IDT array is large while that of the thicker IDT array is small at *f*_s2_. This difference results in the existence of two main Lamb modes and two different resonance frequencies in a DLWR. It is precisely because of this characteristic that these two resonance frequencies can be adjusted separately by changing the thicknesses of the IDTs.

Additionally, the piezoelectric coefficient of ScAlN is larger than that of AlN [27,28], so the resonator has a larger effective electromechanical coupling coefficient, and the bandwidth of the dual-passband filter can meet the design requirements. Molybdenum is chosen as the material of electrode to minimize the acoustic attenuation and to provide good electrical conductivity [29,30]. Moreover, molybdenum also provides a good growth surface for piezoelectric films [31].

In this paper, the LWR comprises 100-nm-thick bottom IDTs, 750-nm-thick Sc_0.2_Al_0.8_N and 100-nm-thick top IDTs. Moreover, a 750-nm-thick Sc_0.2_Al_0.8_N film is also used in the DLWR, while there are IDT arrays with two different thicknesses. The thickness of one array is fixed at 100 nm, while the thickness of the other array is set as 100 nm, 200 nm and 300 nm. Finite Element Analysis (FEA) is used in this paper, and the material constants of Sc_0.2_Al_0.8_N used in the simulation are shown as the following [32,33]:

(1) $$C_{xy}\;=\;\left[\begin{array}{cccccc} 331 & 141 & 122 & 0 & 0 & 0\\ 141 & 331 & 122 & 0 & 0 & 0\\ 122 & 122 & 299 & 0 & 0 & 0\\ 0 & 0 & 0 & 104 & 0 & 0\\ 0 & 0 & 0 & 0 & 104 & 0\\ 0 & 0 & 0 & 0 & 0 & 91 \end{array}\right]$$

(2) $$e_{xy}\;=\;\left[\begin{array}{cccccc} 0 & 0 & 0 & 0 & -0.27 & 0\\ 0 & 0 & 0 & -0.27 & 0 & 0\\ -0.71 & -0.71 & 2.22 & 0 & 0 & 0 \end{array}\right]$$

(3) $$\varepsilon_{xy}\;=\;\left[\begin{array}{ccc} 11.5 & 0 & 0\\ 0 & 11.5 & 0\\ 0 & 0 & 12.4 \end{array}\right]$$

where *C_xy_* is the elasticity matrix, $e_{xy}$ is the coupling matrix, and $\varepsilon_{xy}$ is the relative permittivity.

### 2.2. Simulation Analysis

**Figure 2:**
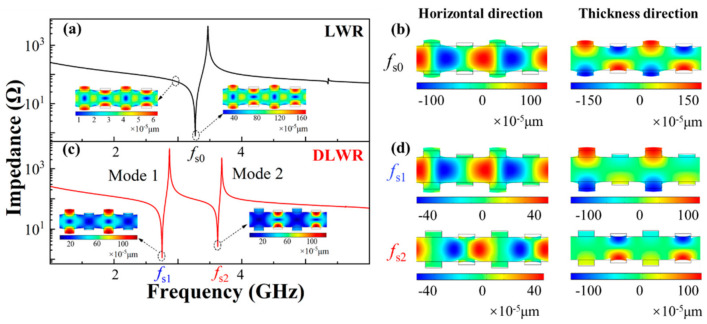
The simulated frequency responses and displacements of LWR and DLWR: (**a**) impedance curve and total displacement of LWR; (**b**) displacements of LWR in horizontal and thickness directions; (**c**) impedance curve and total displacement of DLWR; (**d**) displacements of DLWR in horizontal and thickness directions.

The principle of the DLWR is put forward and verified by FEA simulation. Figure 2 shows the comparison of the displacements and the impedance curves of the LWR and DLWR, which verifies the analysis of the existence of the two main modes in the DLWR.

The difference between the structure of the LWR and DLWR used for simulation is shown in Figure 1. Moreover, the comparison of the total displacements between the LWR and DLWR is shown in Figure 2a,c. There is only one main mode in the LWR, but the second main mode appears in the DLWR, which is consistent with the previous analysis. Moreover, through the analysis of the displacement, the reason for the appearance of the second main mode can be seen more intuitively. In the DLWR, the displacement magnitude at *f*_s1_ of the first IDT array and the displacement magnitude at *f*_s2_ of the second IDT array are both about 1 nm, which is consistent with the max displacement magnitude of the IDTs in the LWR. Moreover, the displacement magnitude at *f*_s1_ of the second IDT array and the displacement magnitude at *f*_s2_ of the first IDT array are roughly equivalent to the max displacement magnitude at the non-resonance frequency of the LWR. Additionally, the comparison of the displacement in the horizontal and thickness directions between the LWR and DLWR is shown in Figure 2b,d. The horizontal propagation of Lamb waves in the DLWR is the same as that of the Lamb waves in the LWR, but the propagation of Lamb waves in the thickness direction is quite different. In the LWR, the displacement magnitude in the thickness direction of all IDTs is coincident. However, the displacement magnitude of the first IDT array in the DLWR is large at *f*_s1_ and is small at *f*_s2_, while the situation of the displacement magnitudes at *f*_s1_ and at *f*_s2_ of the second IDT array is the opposite to those of the first IDT array.

## 3. Simulation Results and Discussion

### 3.1. Optimization of $K_{eff}^{2}$

**Figure 3:**
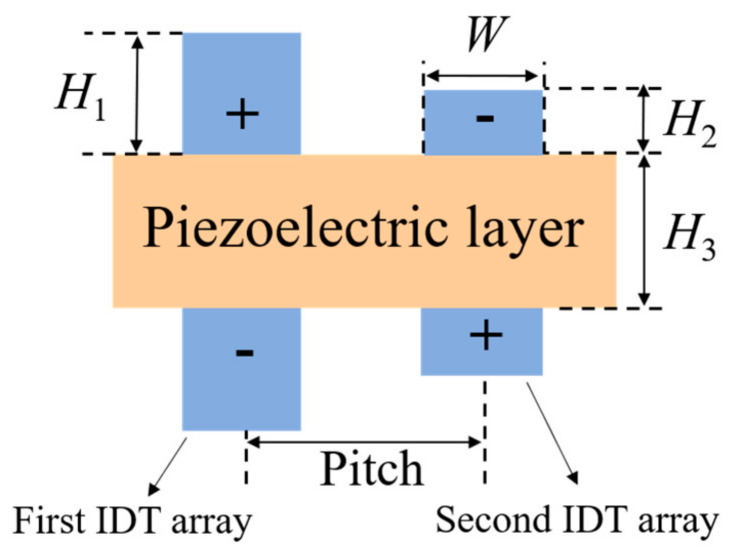
Structural parameters of DLWR.

The structural parameters of the DLWR are shown in Figure 3, where *H*_1_ is the thickness of the IDTs in the first array, *H*_2_ is the thickness of the IDTs in the second array, *H*_3_ is the thickness of the piezoelectric layer, *W* is the width of the IDTs, and pitch is the periodic length. It is worth mentioning that the device is defined as an LWR when *H*_1_ is equal to *H*_2_.

As previously mentioned, the influence of pitch is negligible because the pitch in both the LWR and DLWR affects resonance frequency, $K_{eff}^{2}$, *Q* values and spurious modes. In addition to this, the bandwidth of the filter is closely related to the $K_{eff}^{2}$ of the resonator, so it is very important to study the influence of pitch on the $K_{eff}^{2}$ of resonators [34]. $K_{eff}^{2}$ is used to estimate the energy transduction efficiency between the electrical and mechanical domains in the RF resonators. It is approximately calculated by the following Equation (4) based on the first-order Taylor approximation [35] in this paper:

(4) $$K_{eff}^{2}\;=\;\frac{\pi^{2}}{4}\frac{f_{p}-f_{s}}{f_{p}}$$

**Figure 4:**
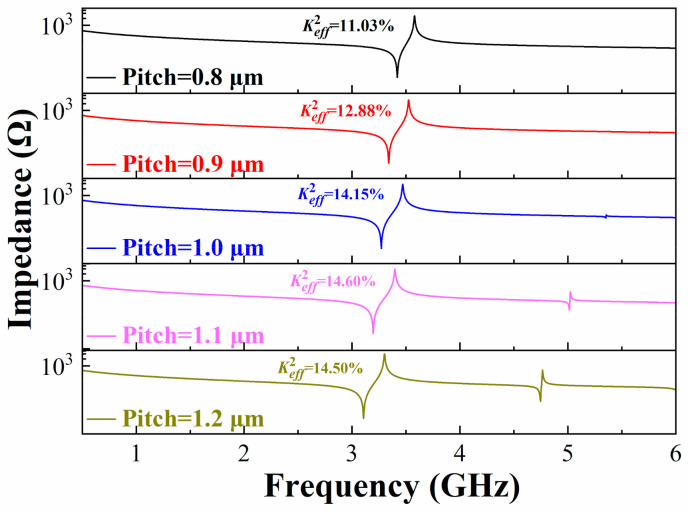
Optimization of the pitch in LWR for high $K_{eff}^{2}$ and elimination of spurious modes.

Figure 4 shows the impedance curves of the LWR with different IDT pitches from 0.8 μm to 1.2 μm when the IDT width is fixed at 0.5 μm. When the pitch is 1.1 μm, $K_{eff}^{2}$ reaches the maximum value of 14.6%, but a spurious mode appears at about 5 GHz. The $K_{eff}^{2}$ of LWR is 14.15% when the pitch is 1.0 μm, and there is no obvious spurious mode in the LWR.

### 3.2. Performance of DLWR

A LWR can be converted to a DLWR when *H*_1_ is different from *H*_2_, and the resonance peak of the second main mode appears in the impedance curve of the DLWR.

**Figure 5:**
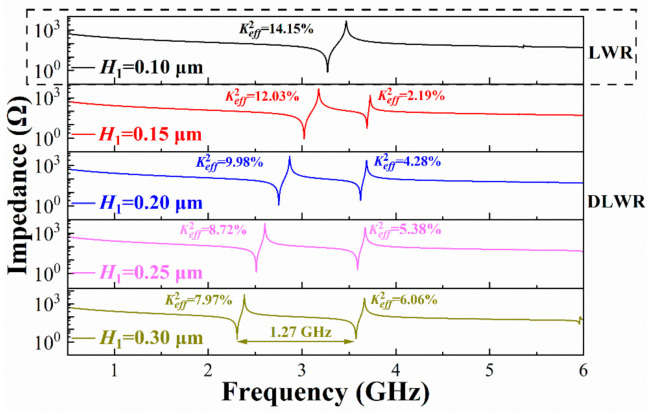
Impedance curves of DLWR with different *H*_1_.

Figure 5 shows the impedance curves of the structure shown in Figure 3. The pitch is fixed at 1.0 μm, and *H*_2_ is fixed at 0.1 μm. The greater difference between *H*_1_ and *H*_2_ leads to the farther distance between the two peaks in the impedance curve. When *H*_1_ is 0.3 μm, the difference of the resonance frequency can reach up to 1.27 GHz.

**Figure 6:**
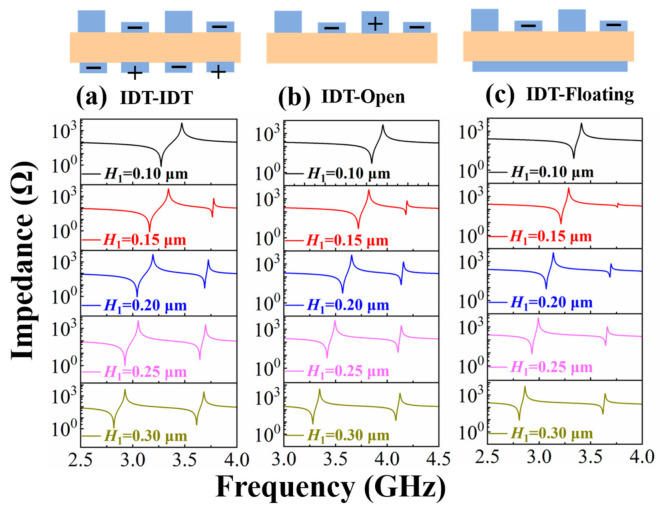
Impedance curves of three other DLWR structures with different bottom electrodes: (**a**) IDT–IDT structure with one bottom IDT array, (**b**) IDT-Open structure without bottom electrode and (**c**) IDT-Floating structure with a floating metal plate.

In addition to the structure shown in Figure 3, we also studied other structures. Figure 6 shows the impedance curves of DLWRs with three other IDT configurations, and *H*_1_ is set from 0.1 μm to 0.3 μm. The characteristics of the impedance curves corresponding to the three different designs are similar to the results in Figure 5.

**Figure 7:**
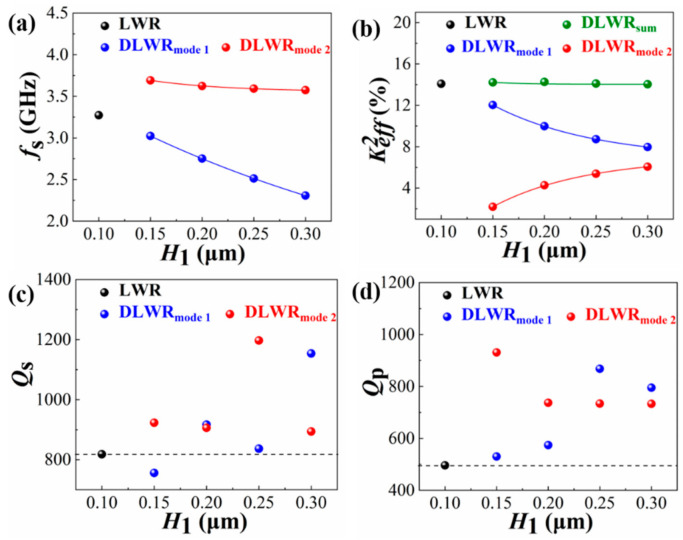
Influences of *H*_1_ on performance of resonators, including (**a**) *f*_s_, (**b**) $K_{eff}^{2}$, (**c**) *Q*_s_ and (**d**) *Q*_p_. DLWR_sum_ is the sum $K_{eff}^{2}$ of mode 1 and mode 2.

The IDT–IDT structure with two bottom IDT arrays shown in Figure 3 is used to further analyze the performance of the DLWRs. Figure 7 shows the *f*_s_, $K_{eff}^{2}$, *Q*_s_ and *Q*_p_ values of the resonators with different *H*_1_ when *H*_2_ is fixed at 0.1 μm. *Q*_s_ and *Q*_p_ represent Q at resonance frequency and anti-resonance frequency, respectively. The resonance frequencies of the two main modes (*f*_s1_ and *f*_s2_) appear in Figure 7a when *H*_1_ differs from *H*_2_. As *H*_1_ increases, *f*_s1_ is significantly reduced. The *f*_s1_ can be adjusted to the frequency designed by changing *H*_1_. In addition, *f*_s2_ can be adjusted in the same way.

Figure 7b shows the influence of the IDT thickness on $K_{eff}^{2}$. When *H*_1_ increases, the $K_{eff}^{2}$ of the first main mode decreases and that of the second main mode increases. However, it is worth noting that the sum of the two $K_{eff}^{2}$ values in the DLWR is almost constant when *H*_1_ changes. The sum of $K_{eff}^{2}$ in the DLWR is equal to the $K_{eff}^{2}$ of the LWR with the same *W* and pitch. In addition, the *Q*_s_ and *Q*_p_ values of the LWR and DLWR are shown in Figure 7c,d. The *Q* value is calculated using the 3 dB bandwidth method, which represents a trend of change [36]. There is no obvious law for the change in the *Q* value. Although the thickening of the electrode reduces the electrical loss, the change in vibration mode shown in Figure 2 also affects the mechanical loss of the resonator.

**Figure 8:**
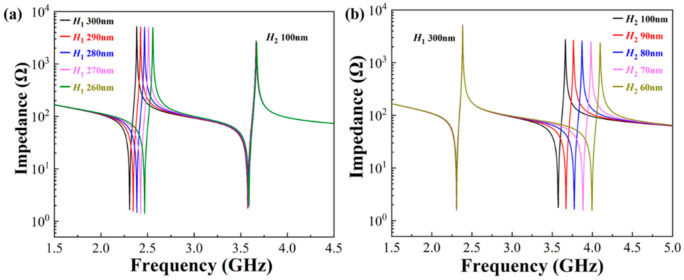
Influences of (**a**) *H*_1_ and (**b**) *H*_2_ on resonance frequencies of the two main modes in DLWRs.

Adjusting the resonance frequencies of the two main modes without interference is very important for dual-passband filters. Figure 8 shows the influence of the thicknesses of the IDTs on the resonance frequencies. When *H*_2_ is fixed and *H*_1_ changes, *f*_s1_ alters accordingly. Correspondingly, when *H*_1_ is fixed and *H*_2_ changes, *f*_s2_ alters. DLWRs with two adjustable resonance frequencies can be achieved by changing the thicknesses of the IDTs. This advantage of DLWRs provides favorable conditions for the construction of adjustable dual-passband filters.

## 4. Design of Dual-Passband Filter

**Figure 9:**
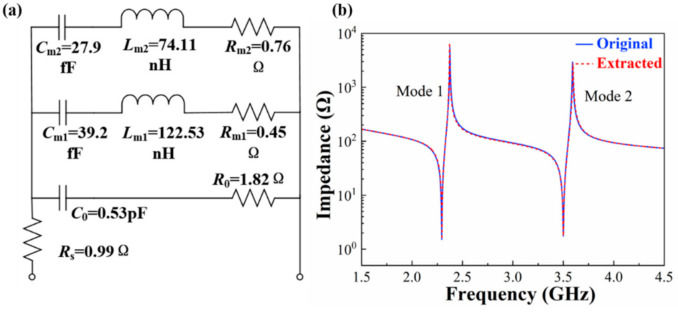
(**a**) MBVD model with two motional branches. (**b**) Simulated and MBVD fitting results of DLWR.

The two modes in the DLWR can be characterized by the modified Butterworth–Van Dyke (MBVD) fitting model [37]. As shown in Figure 9a, the model consists of a static capacitor (*C*_0_), dielectric loss (*R*_0_), electrode resistance (*R*_s_) and two motional branches corresponding to mode 1 (*R*_m1_, *L*_m1_, *C*_m1_) and mode 2 (*R*_m2_, *L*_m2_, *C*_m2_). *C*_0_, *R*_0_, *R*_s_ and six motional elements can be calculated by *f*_s_, *f*_p_ and three impedance values (*Z*_1_, *Z*_2_, *Z*_3_) using Equations (5)–(10):

(5) $$\gamma \;=\;\frac{1}{{\left(\frac{f_{p}}{f_{s}}\right)}^{2}-1}\;=\;\frac{C_{0}}{C_{m}}$$

(6) $$Im\left(Z_{1}\right)\;=\;\frac{1}{2\pi f_{s}\left(1+\frac{1}{\gamma }\right)C_{0}}$$

(7) $$f_{s}\;=\;\frac{1}{2\pi \sqrt{L_{m}C_{m}}}$$

(8) $$Re\left(Z_{1}\right)\;=\;\frac{\gamma^{2}R_{0}+R_{m}}{{\left(1+\gamma \right)}^{2}}+R_{s}$$

(9) $$Re\left(Z_{2}\right)\;=\;R_{s}+R_{m}$$

(10) $$Re\left(Z_{3}\right)\;=\;\frac{1}{{\left(2\pi f_{p}C_{0}\right)}^{2}\left(R_{0}+R_{m}\right)}$$

where γ is the ratio of $C_{0}$ and $C_{m}$. *Z*_1_ is the impedance at the non-resonance frequency. *Z*_2_ and *Z*_3_ are the impedance at *f_s_* and *f_p_*, respectively. The *f_s_*, *f_p_* and impedance values of the two modes are used to calculate the motional elements of the two resonance peaks.

Figure 9b shows the results of the FEA simulation and fitness using the MBVD model. The blue curve is the simulated result of the DLWR, and the red one is the MBVD fitting curve. The MBVD fitting curve shows great concordance with the simulated result for both of the two modes.

The resonance frequency and the $K_{eff}^{2}$ of the DLWR depend on the pitch and the two thicknesses of the IDTs when the thickness of the piezoelectric film is fixed. The resonance frequencies and the $K_{eff}^{2}$ of the two modes in the DLWR determine the center frequencies and the bandwidth of the DLWR-based dual-passband filters, respectively. There are dozens of RF filters in the RF front-end module, and they support different frequency bands. Among these important frequency bands, we chose Wi-Fi 2.4 G and n77 as our target frequency bands for design. We use two sets of resonators with optimized structural parameters as shown in Table 1 to design the dual-passband filter. The W is fixed at 0.5 μm, and a pitch of 1.05 μm is chosen in the design.

**Figure 10:**
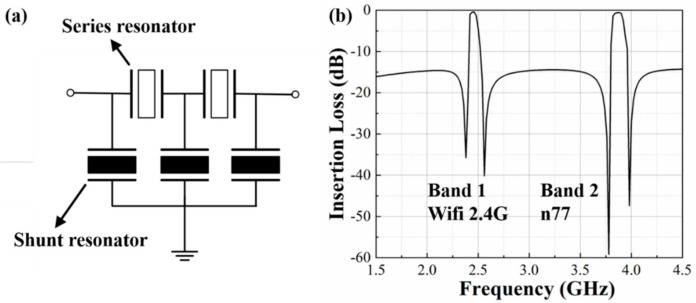
Schematic circuit design and simulated transmission response of the dual-band filter: (**a**) ladder topology of the dual-band filter; (**b**) insertion loss of the filter.

A ladder configuration of the dual-passband filter used in the simulation is shown in Figure 10a. It consists of two series DLWRs and three shunt DLWRs. Figure 10b shows the frequency response of the dual-band filter with two center frequencies at 2.45 GHz and 3.84 GHz, and the bandwidths are 66 MHz and 112 MHz, respectively. The filter is demonstrated with an insertion loss less than 1 dB and a band rejection of about 15 dB. The simulated result in Figure 10 verifies the potential feasibility of dual-passband filters by using dual-mode DLWRs.

## 5. Conclusions

In this paper, we present a dual-mode Lamb wave resonator (DLWR) and a dual-passband filter using the DLWRs. The influences of IDT thicknesses and IDT pitch in the DLWR on resonance frequencies, *Q* values, $K_{eff}^{2}$ and spurious modes are investigated by a theoretical analysis and FEA simulation. The resonance frequencies and the $K_{eff}^{2}$ of the DLWRs strongly depend on the pitch and the thicknesses of the IDTs. A resonator without an obvious spurious mode can be obtained by optimizing pitch, and a high $K_{eff}^{2}$ can also be achieved, which leads to a large bandwidth of the filter. The impedance curve of the resonator shows no spurious mode, and a $K_{eff}^{2}$ of 14.15% is obtained when the width of the IDTs is 0.5 μm and the pitch is 1 μm. In the DLWR, when the thickness of the IDT in the first IDT array is 0.3 μm and the thickness of the IDT in the second array is 0.1 μm, *f*_s1_ is 2.32 GHz and *f*_s2_ is 3.59 GHz, respectively. In addition to this, we demonstrate a dual-passband filter with an insertion loss of less than 1 dB and a band rejection of about 15 dB based on optimized DLWRs. Two passbands at 2.45 GHz and 3.88 GHz with the bandwidths of 66 MHz and 112 MHz, respectively, are achieved. The resonance frequencies of the two main modes are strongly related to the thicknesses of the IDTs and can be adjusted separately, which potentially satisfies the requirements of dual-passband filters for 5G communications.

## Figures and Tables

**Table 1:** Parameters of series resonator and shunt resonator.

Resonator	Pitch (μm)	*W* (μm)	*H*_1_ (μm)	*H*_2_ (μm)	*f_s_*_1_ (GHz)	*f_p_*_1_ (GHz)	*f_s_*_2_ (GHz)	*f_p_*_2_ (GHz)
Series	1.05	0.5	0.260	0.066	2.464	2.552	3.884	3.976
Shunt	1.05	0.5	0.277	0.175	2.392	2.476	3.785	3.879

## Data Availability

Data is contained within the article.
